# Probabilistic Learning Coherent Point Drift for 3D Ultrasound Fetal Head Registration

**DOI:** 10.1155/2020/4271519

**Published:** 2020-01-31

**Authors:** Jorge Perez–Gonzalez, Fernando Arámbula Cosío, Joel C. Huegel, Verónica Medina-Bañuelos

**Affiliations:** ^1^Instituto de Investigaciones en Matemáticas Aplicadas y en Sistemas, Universidad Nacional Autónoma de México, Mérida, Yucatán, Mexico; ^2^Biomechatronics Laboratory, School of Engineering and Science, Tecnologico de Monterrey, Guadalajara, Mexico; ^3^Center for Extreme Bionics, Massachusetts Institute of Technology, Cambridge, MA, USA; ^4^Neuroimaging Laboratory, Electrical Engineering Department, Universidad Autónoma Metropolitana, Iztapalapa, Mexico

## Abstract

Quantification of brain growth is crucial for the assessment of fetal well being, for which ultrasound (US) images are the chosen clinical modality. However, they present artefacts, such as acoustic occlusion, especially after the 18^th^ gestational week, when cranial calcification appears. Fetal US volume registration is useful in one or all of the following cases: to monitor the evolution of fetometry indicators, to segment different structures using a fetal brain atlas, and to align and combine multiple fetal brain acquisitions. This paper presents a new approach for automatic registration of real 3D US fetal brain volumes, volumes that contain a considerable degree of occlusion artefacts, noise, and missing data. To achieve this, a novel variant of the coherent point drift method is proposed. This work employs supervised learning to segment and conform a point cloud automatically and to estimate their subsequent weight factors. These factors are obtained by a random forest-based classification and are used to appropriately assign nonuniform membership probability values of a Gaussian mixture model. These characteristics allow for the automatic registration of 3D US fetal brain volumes with occlusions and multiplicative noise, without needing an initial point cloud. Compared to other intensity and geometry-based algorithms, the proposed method achieves an error reduction of 7.4% to 60.7%, with a target registration error of only 6.38 ± 3.24 mm. This makes the herein proposed approach highly suitable for 3D automatic registration of fetal head US volumes, an approach which can be useful to monitor fetal growth, segment several brain structures, or even compound multiple acquisitions taken from different projections.

## 1. Introduction

Fetal ultrasound (US) is the most commonly used imaging modality in obstetrics because it does not require ionizing radiation, works in real time, the transducer is easily manipulated, and is inexpensive compared to other imaging systems such as Computed Tomography (CT) or Magnetic Resonance Imaging (MRI). It provides valuable information of the fetal central nervous system, which presents some of the most relevant structures for fetal clinical assessment [1]. However, US images present some drawbacks, such as speckle noise, intensity variations dependent on the patient and operator, and acoustic shadows. The phenomenon of acoustic occlusion occurs mainly in the second and third trimesters of pregnancy due to nonuniform fetal cranial calcification, which affects the acoustic beam penetration of ultrasound waves; it may impact on the quality of fetal brain images because they can present acoustic shadows and missing tissue information [1]. This phenomenon can cause difficulty in adequate measurement of several important brain structures such as cerebellum or nuchal translucency, which are used for the detection of several maternal-fetal diseases or for weight estimation at birth.

According to the International Society of Ultrasound in Obstetrics and Gynecology [2], there are different acquisition planes depending on the fetal brain structures to be analyzed, the main being axial, sagittal, and coronal. Each plane is obtained by changing the acquisition angle and can provide information about brain tissue that may be complementary due to the phenomenon of nonuniform acoustic occlusion in the fetal head. Currently, in clinical practice, each plane is used individually depending on the area to be analyzed, which could be a limiting factor in the measurement of brain indices and in clinical assessment. In this work, a new automatic rigid registration method of several 3D fetal brain views is proposed. This can be useful to compare fetal growth in different stages of pregnancy, as a tool to segment different structures or to combine fetal brain information from different views in order to attend acoustic occlusion artifacts [3, 4]. The method presented focuses on modeling the fetal cranium as a point cloud and performing an automatic alignment using a Coherent Point Drift (CPD) approach, weighted by the membership probability of each point obtained *a priori* with a random forest classifier.

The alignment or registration of a set of volumes is a mathematical approach that consists of finding an optimal geometric transformation *𝒯* which maps each different volume to a common reference view. Most of the methods of registration are based on intensity or geometrical features [5]. The former seeks to match the intensity levels of two volumes by a similarity measure. Among the main metrics are mutual information (MI), cross correlation (CC), and mean square error (MSE), that quantify the joint information between the intensity levels of the two volumes to be aligned [6–9]. However, these algorithms can be computationally expensive and are sensitive to intensity variations or missing data.

On the other hand, geometric or point set correspondence registration methods are based on automatically or semiautomatically aligning distinctive features (contours, intersections, or corners) of an image or volume. Like in intensity-based methods, the problem is to find an optimal transformation matrix, which registers a displaced volume to the same coordinate space of a reference volume, by maximizing the correspondence between a set of points or salient features that describe both volumes. The point cloud registration approaches have several applications in computer vision, pattern recognition, object detection, pose estimation, medical image analysis, modeling, and feature extraction [10]. These algorithms present several advantages: they have lower computational cost compared to intensity-based methods, they can be minimally sensitive to missing data or outliers, and because they do not consider information intensity, they are immune to additive or multiplicative noise, which are common artefacts in US fetal images. However, these alignment approaches require a point cloud that describes the enveloping area of the volumes to register or an optimal detection of salient features. According to Savva et al. [11], intensity-based registration methods are computationally more expensive compared to geometry-based methods; however, the latter show better performance when registering key points, point clouds, or envelope surfaces.

Several point set registration methods have been reported such as iterative closest point (ICP) [12, 13], which is based on establishing correspondence of pairs of points by iteratively minimizing the mean square error of the distance between them; Random Sample Consensus (RANSAC) [14] which is based on the iterative adjustment of a model or Scale-Invariant Feature Transform (SIFT) that uses multiple Gaussian filters to find the salient points needed in the alignment process [15]. However, to perform a proper alignment these algorithms require well-defined outlines, lines or salient points, and characteristics that are not present in US fetal images.

In contrast, Coherent Point Drift (CPD) proposed by Myronenko and Song [16] is a robust computationally fast algorithm that achieves good results using point clouds with outliers, noise, and missing data and outperforms most state-of-the-art geometric-based approaches. These characteristics make CPD a suitable method to register sets of points extracted from US fetal brain volumes because, as mentioned before, cerebral acoustic occlusion and speckle noise in US images are crucial factors to be considered to carry out an adequate alignment.

Given a set of points to be aligned and a second point cloud modeled as a set of centroids of a Gaussian mixture model (GMM), the CPD method registers both point clouds by iteratively searching for an optimal transformation matrix *𝒯*. Some of the most notorious disadvantages of the CPD method are that an equiprobable membership of GMM is considered, which can affect the registration process because not all points contribute equally. In addition, it considers an isotropic covariance matrix of the GMM to ensure that the algorithm converges. Finally, it incorporates a user-defined weight factor *w* on the GMM to deal with outliers, noise, and missing data. For these reasons, several researchers have proposed variants on the CPD method.

Wang et al. [17] propose a search method that combines genetic algorithms with Nelder–Mead simplex approach to automatically define the weight factor *w*. Their results show a reduction of up to 50% error in cases with a high number of outliers. Gao et al. [18] designed a method to update the weight factor *w* while the CPD algorithm iteratively aligns two sets of points. This is achieved by incorporating the factor *w* in the transformation parameters optimization. Their results show robustness against noise and outliers. Liu et al. [19] reported an Automatic Outlier Suppression (AOS) algorithm for the CPD method. It is inspired by bidirectional normalizations of the matrix and outlier rejection in the probabilistic relaxation labeling algorithm, which can provide robust point matching by preserving local neighborhood structures. de Sousa and Kropatsch [20] implemented a variant of CPD by integrating centrality measures such as degree, betweenness, closeness, eigenvector, and pageRank centralities using Delaunay triangulation. These metrics are indicators of the topological relationships between neighboring points and were incorporated independently into a probabilistic cost function. The results show improvements in the CPD method; however, it is necessary to combine these indices to achieve better performance. Zhang et al. [21] incorporated SURF as a preliminary step to align contours; afterwards, the membership probability of the GMM is estimated by the edge point confidence. This CPD variant was applied to synthetic aperture radar images obtaining better results compared to the original method. Lu et al. [22] proposed the accelerated CPD for fast registration of 3D point clouds. These authors incorporated global squared iterative expectation-maximization algorithm with fast Gauss transform to the CPD method, managing to reduce the processing time up to ten times. Other authors have reported CPD variants seeking to optimally assign the GMM membership probability using rotation invariant shape descriptors [23], correspondence priors and correspondence preserving subsampling approaches [24], and the shape context of one point with respect to the distribution of other points [25]. On the other hand, Saval-Calvo et al. [26] proposed color-CPD algorithm to register 3D points by using color and shape spaces to jointly estimate the best match. These authors consider that the incorporation of color helps to register sets of points with noise or missing data. The color-CPD combines the color information in the iterative process of aligning two sets of points. However, this method considers an isotropic covariance matrix and an equal membership probability in the GMM. In general, these methods report variations of the CPD algorithm, seeking to improve the computational speed, the automatic weighting of the membership probability, and the weight factor definition to deal with outliers and missing data for different applications of 2D and 3D image registration. A disadvantage to highlight is that these methods need an initial point cloud prior to registration, which is not available in US fetal brain studies because they are constituted by intensity voxels.

For the specific application of 3D US fetal head alignment, several works have been published. This is a difficult task due to multiplicative noise, fetal movement and acoustic shadows, and factors that hinder an adequate alignment. In this context, Cen et al. [27, 28] have worked on the registration of volumes using shape and texture patterns extracted from a Gabor filter bank. This set of features was used to register studies in 2D and 3D using only one fetal brain. Fathima et al. [29] reported a method based on representing each image by amplitude, orientation, and local phase. It is used to perform the affine registration based on normalized mutual information. Chen et al. [30] proposed an algorithm for fetal head registration of US phantom volumes. It is based on previously matching segmented features such as the eyes or the fetal head using feature-based registration.

In other image modalities, Kuklisova et al. [31] reported a registration method between fetal brain MRI and US studies, in which the former was converted into a pseudovolume of US using a probabilistic atlas. Subsequently, the pseudovolume is aligned towards a conventional US fetal brain study using a robust block-rigid registration. The aim of this work was to use the information from the registered MRI study to improve the visualization of fetal brain anatomical structures. All these works, seek to register fetal brain studies of US-US and US-MRI in two and three dimensions. However, some of them use synthetic images or phantoms. Additionally, in none of the cases do they consider studies with artefacts of fetal brain occlusion, which considerably affects the registration methods based on intensity or texture.

An exhaustive review of US image registration was published by [32]. The authors include image-based methods, such as MI, CC, or MSE; correspondence-based methods, where characteristics were selected either manually or using algorithms like SIFT; or other similarity measures, such as Hellinger distance, statistics-based fuzzy local binary patterns, or the sum of absolute differences. According to this review, the most important drawbacks for US registration are multiplicative speckle and occlusion artefacts, while the main reported medical imaging applications are in head, neck, breast, heart, liver, kidney, bone, prostate, and fetal studies.

To the best of our knowledge, there are not any research studies that address the problem of automatic rigid registration of 3D US fetal heads based on point clouds that can work correctly in the presence of artefacts such as multiplicative noise, occluded brain regions, and outliers.

In the present work, a registration strategy is proposed that has two main contributions:

1. Automatic registration of real 3D US fetal brain volumes contains a considerable degree of occlusion artefacts, noise, and missing data. Previous research studies have not addressed this issue in a real clinical context. As already mentioned, the alignment of fetal studies can be useful in several relevant clinical applications, such as brain structures segmentation, fetal growth monitoring, multiprojection fusion, or acoustic shadows mitigation.
2. A variation of the CPD method that incorporates new features: automatic segmentation and conformation of the point cloud, qualities that have not been reported by any previously developed CPD variant, together with an appropriate proposed weighting of membership probabilities of the GMM. Segmentation as well as weighting factors are obtained from an RF-based classifier, fed by a set of features composed of intensity, texture, and edge parameters. Preliminary results of this research have been reported in [33], in which a variant of the CPD method using another scheme of weighting was presented.

In Section 2, the registration methodology as well as the characteristics of the fetal studies used for training and validation is described in detail. In Section 3, the obtained results, the corresponding discussion, and the comparison against other intensity-based and geometric-based registration methods are exposed. Finally, in Section 4, the conclusions drawn from this work are presented.

## 2. Material and Method

**Figure 1:**
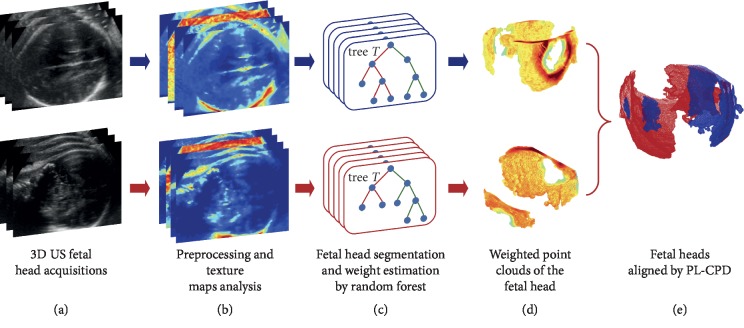
Methodological diagram.

The overall diagram of the proposed methodology is shown in Figure 1 and will be described in detail in the following sections. It starts with the acquisition of multiple US volumes of the fetal head observed at different projections (Figure 1(a), Section 2.1) followed by a preprocessing step where confidence maps and texture features are computed (Figure 1(b)). These parameters are used for segmentation purposes and to estimate probabilistic weights by means of Random Forest classification (Figure 1(c), Section 2.2). The point clouds built from the segmented fetal head are then weighted with the posterior probability values estimated from the Random Forest process (Figure 1(d), Section 2.2) to finally carry out the registration between the two point clouds, using the proposed method (Figure 1(e)), as described in Section 2.3.

### 2.1. Fetal Brain Data Set

Eighteen fetal brains between 20 and 24 weeks of gestational age were acquired using a curvilinear ultrasound transducer in mode B, at frequencies of 8–20 MHz (Voluson E8, General Electric Healthcare Company, USA). All volumes had isotropic resolutions going from 0.2 to 0.5 mm^3^. Each case consisted on two studies, taken at coronal and axial projections to the same patient in equal conditions. All studies were acquired by obstetrics experts in Fetal Medicine and were approved by the Department of Fetal Medicine of the National Institute of Perinatology (INPer). All patients gave their consent according to the Declaration of Helsinki. Finally, each pair of volumes was manually aligned by experts in obstetrics from INPer; for this, several fiduciary points were considered such as middle line, peduncles, and thalamus, among others. This step will be important for the evaluation and comparison of the proposed registration method.

### 2.2. Random Forest Probabilistic Weights

As a preprocessing step, the confidence map was calculated for each US acquisition. The purpose is to emphasize shaded or attenuated regions in US data obtaining images with homogeneous intensities. It has been proven that these maps can help in the processing and registration of US data [34].

Subsequently, a binary classification scheme based on Random Forest (RF), which is a machine learning algorithm based on the ensemble of multiple decision trees [35], was implemented to find the weighting factors. In a previous research, a Support Vector Machine (SVM) classifier was tested for this purpose [33], with similar performance but with a higher computational cost.

**Figure 2:**
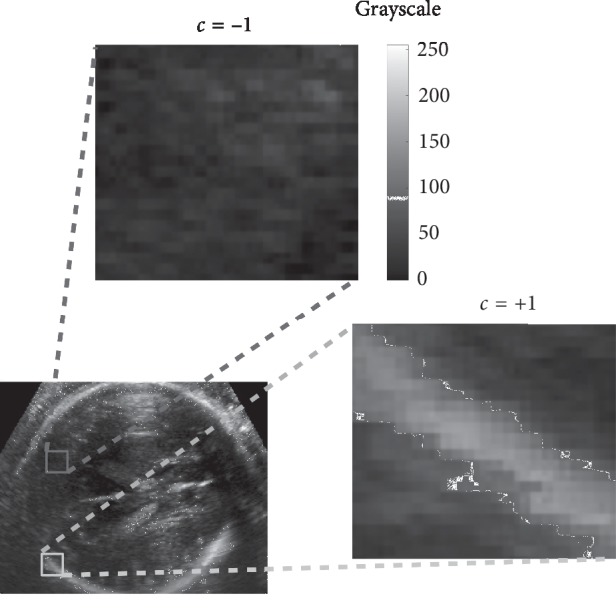
Classes considered for fetal cranium segmentation.

Given a training data set **V**=(*x*_1_, *x*_2_,…, *x*_*d*_) ∈ **R**^*d*^ belonging to two classes {*c*=+1, *c*=−1}, where **V** represents each voxel, *x*_*d*_ are the responses of a chosen filter bank at that particular voxel, and *d* is the feature number or extracted patterns in each voxel. The task is to optimize an energy function through the training set *S*_0_ (root node), associated with a labeled data set. The optimization function can be defined as follows:

(1) $$\begin{array}{c} \beta_{j}=\underset{\beta \in \psi_{j}}{\arg\;\max}I\left(S_{j},\theta \right), \end{array}$$

where for each subset of input training data *S*_*j*_, RF learns the function that best splits *S*_*j*_ into left *S*_*j*_^*L*^ and right *S*_*j*_^*R*^ nodes. In this context, *j* represents each division node, *β* is a set of split parameters, and *ψ*_*j*_ is a random subset of the entire parameter space *ψ*. These parameters help to reduce possible overfitting thus improving the generalization capabilities of RF. For classification purposes the function *I* can be defined as the gain information for discrete distributions:

(2) $$\begin{array}{c} I\left(S_{j},\beta \right)=H\left(S_{j}\right)-\underset{i\in \left\{L,R\right\}}{\sum }\frac{\left|S_{j}^{i}\right|}{\left|S_{j}\right|}H\left(S_{j}^{i}\right), \end{array}$$

with *i* indexing the two child nodes (left *L* or right *R* node) and *H*(*S*_*j*_^*i*^) being the entropy of a given subset *S*_*j*_^*i*^, which can be defined as follows:

(3) $$\begin{array}{c} H\left(S_{j}^{i}\right)=-\underset{c\in \pm 1}{\sum }P\left(c\right)\log\;P\left(c\right), \end{array}$$

where *p*(*c*) is calculated as the normalized empirical histogram of labels corresponding to the training points in *S*_*j*_^*i*^. To assemble the result of all the decision trees, given the class *p*(*c*=+1|**V**), the content result of each leaf is accumulated by

(4) $$\begin{array}{c} P\left(\left.c=+1\right|V\right)=\frac{1}{T}\overset{T}{\underset{t=1}{\sum }}P_{t}\left(\left.c=+1\right|V\right), \end{array}$$

where *t*={1,2,…, *T*} represents the number of trees used in the classification and *P*(*c*=+1|**V**) is denoted as the *posterior probability* of the classification process. For this work, the fetal cranium is considered as class *c*=+1 (segmentation required to build the point cloud) and the rest of brain tissue and acoustic artefacts as class, as it can be seen in Figure 2.

The task is to automatically classify these two classes using a set of features that includes intensity information and several texture filters such as variance, rank, entropy, median, and Wiener estimated on an (9 × 9 × 9) analysis window [36]. Other incorporated features were the edges obtained from Canny [37] and Laplacian filtering. Following the fetal cranium segmentation, the point cloud is constructed using constrained Delaunay tetrahedralization [38]. The point clouds are the vertices in cartesian coordinates of the obtained triangular mesh and correspond to the inner and outer enveloping areas of the fetal cranium.

### 2.3. Probabilistic Learning Coherent Point Drift

The method proposed for fetal brain volume registration is based on the algorithm developed by Myronenko and Song [16] known as Coherent Point Drift. It consists of a geometric registration that establishes point to point correspondences and assumes that each element drifts coherently as a group, while preserving the points' cluster topology. Let *X* and *Y* be two sets of points to be registered. They can be denoted as *Y*={*y*_*m*_|1,2,…, *M*} and *X*={*x*_*n*_|1,2,…, *N*}, where *Y* corresponds to the set of centroids of the GMM and *X* is the generated points' set. The task is to obtain an affine transform *𝒯*, such that *X*=*𝒯*(*Y*, Θ), where Θ is a matrix containing rotation, translation, and scaling parameters. The probability density function (PDF) on a GMM can be written as follows:

(5) $$\begin{array}{c} p\left(x\right)=\overset{M+1}{\underset{m=1}{\sum }}P\left(m\right)p\left(\left.x\right|m\right), \end{array}$$

where

(6) $$\begin{array}{c} p\left(\left.x\right|m\right)=\frac{1}{2\pi \sigma^{2}}\exp\left\{-\frac{{\left|\left|x-T\left(y_{m},\theta \right)\right|\right|}^{2}}{2\sigma^{2}}\right\},\;m=1,2,\ldots ,M, \end{array}$$

and *P*(*m*)=(1/*M*) is an equiprobable distribution.

A uniform distribution, denoted by *p*(*x*|*M*+1)=1/*N*, is used to deal with outliers, occlusion artefacts, and noise. A weight factor (0 < *w* < 1) representing the outliers' ratio is incorporated into the distribution, together with an isotropic variance *σ*^2^ term. Therefore, the PDF is formulated as follows:

(7) $$\begin{array}{c} p\left(x\right)=\omega \frac{1}{N}+\left(1-\omega \right)\overset{M+1}{\underset{m=1}{\sum }}\frac{1}{M}p\left(\left.x\right|m\right). \end{array}$$

Parameters' matrix Θ is derived from the GMM centroids and can be estimated by probability maximization or by minimization of the negative logarithmic probability of the following function:

(8) $$\begin{array}{c} E\left(\Theta ,\sigma^{2}\right)=-\overset{N}{\underset{n=1}{\sum }}\log\overset{M+1}{\underset{m=1}{\sum }}P\left(m\right)p\left(\left.x_{n}\right|m\right), \end{array}$$

previously assuming that each gaussian component is independent.

Given two points sets (*y*_*m*_ and *x*_*n*_), the GMM posterior probability is denoted by *p*(*m*|*x*_*n*_)=*P*(*m*)*p*(*x*_*n*_|*m*)/*p*(*x*_*n*_). The CPD method applies an Expectation-Maximization (EM) algorithm to adjust Θ and *σ*^2^ using Bayes' theorem. The posterior probabilities of the mixture components *P*^old^(*m*|*x*_*n*_) (equation (9)) are iteratively computed in, what is known as, the expectation or E-step:

(9) $$\begin{array}{c} P^{\text{old}}\left(\left.m\right|x_{n}\right)=\frac{\exp\left\{-\left({\left|\left|x_{n}-T\left(y_{m},\theta \right)\right|\right|}^{2}/2\sigma^{2}\right)\right\}}{\sum_{k=1}^{M}\exp\left\{\left(-{\left|\left|x_{n}-T\left(y_{m},\theta \right)\right|\right|}^{2}/2\sigma^{2}\right)\right\}+\left(\omega M{\left(2\pi \sigma^{2}\right)}^{D/2}/\left(1-\omega \right)N\right)},\;m=1,2,\ldots ,M. \end{array}$$

The *“new”* parameter values are estimated by minimizing expectation of the complete negative log-likelihood function:

(10) $$\begin{array}{c} Q=-\overset{N}{\underset{n=1}{\sum }}\overset{M+1}{\underset{m=1}{\sum }}P^{\text{old}}\left(\left.m\right|x_{n}\right)\log\left[P^{\text{new}}\left(m\right)p^{\text{new}}\left(\left.x_{n}\right|m\right)\right], \end{array}$$

during the maximization or *M*-step. The new Θ and *σ*^2^ parameters are obtained by rewriting equation (10):

(11) $$\begin{array}{c} Q\left(\Theta ,\sigma^{2}\right)=\frac{1}{2\sigma^{2}}\overset{N}{\underset{n=1}{\sum }}\overset{M+1}{\underset{m=1}{\sum }}P^{\text{old}}\left(\left.m\right|x_{n}\right){\left\|x_{n}-T\left(y_{m},\theta \right)\right\|}^{2}+\frac{DN_{p}}{2}\log\;\sigma^{2}, \end{array}$$

where *D* corresponds to data dimensionality. The algorithm alternatively iterates *E*-step and *M*-step until *Q*'s convergence, thus aligning the sets of points with *X*=*𝒯*(*Y*, Θ).

However, assigning the same probability *P*(*m*)=(1/*M*) to all GMM components can affect registration quality, given that each Gaussian contributes differently in the model. In this work, it is proposed to weight the membership probabilities with the values obtained from the posterior probability resulting from the RF classification process. At the same time, the fetal cranium classification helps to obtain the point clouds used in the registration.

In this way, the GMM membership probabilities *P*(*m*) correspond to the posterior probability (normalized between 0 and 1) obtained during the classification step *P*(*c*=+1|*x*_*n*_) (equation (4)) (where *c*=+1 is the class corresponding to fetal cranium), for each point *x*_*n*_. It can be expressed by

(12) $$\begin{array}{c} P{\left(m\right)}^{*}=\frac{P\left(\left.c=+1\right|x_{n}\right)}{\lambda }, \end{array}$$

where

(13) $$\begin{array}{c} \lambda =\overset{M}{\underset{k=1}{\sum }}P{\left(\left.c=+1\right|x_{n}\right)}_{k}. \end{array}$$

Therefore, *P*^old^(*m*|*x*_*n*_) and the *Q* objective function can be rewritten as follows:

(14) $$\begin{array}{c} P^{\text{old}*}\left(\left.m\right|x_{n}\right)=\frac{P\left(\left.c=+1\right|x_{n}\right)\exp\left\{-\left({\left\|x_{n}-T\left(y_{m},\theta \right)\right\|}^{2}/2\sigma^{2}\right)\right\}}{\sum_{k=1}^{M}P{\left(\left.c=+1\right|x_{n}\right)}_{k}\exp\left\{-\left({\left|\left|x_{n}-T\left(y_{m},\theta \right)\right|\right|}^{2}/2\sigma^{2}\right)\right\}+\left(\omega M{\left(2\pi \sigma^{2}\right)}^{D/2}/\left(1-\omega \right)N\right)},\;m=1,2,\ldots ,M,\\ Q^{*}=Q+\overset{N}{\underset{n=1}{\sum }}\overset{M}{\underset{m=1}{\sum }}P^{\text{old}}\left(\left.m\right|x_{n}\right)\log\;P{\left(m\right)}^{*}. \end{array}$$

With this weighting strategy outliers will have less contribution compared to voxels that describe the fetal cranium, which will have a larger weight during the registration process. These changes do not affect the previously described EM optimization procedure. Thus, the Probabilistic Learning Coherent Point Drift (PL-CPD) method has the advantages of being less sensitive to outliers and do not requiring a high computational cost, compared to other intensity-based methods.

### 2.4. Evaluation of the PL-CPD Method

To assess RF's performance in head segmentation, a 5-fold crossvalidation was carried out, where the training and test data sets were obtained from a sample of five US volumes (an example of the classes is presented in Figure 2). As segmentation performance metric, the Dice index was computed, which is defined as follows:

(15) $$\begin{array}{c} \text{Dice}=\frac{2\left|A\cap B\right|}{\left|A\right|+\left|B\right|}, \end{array}$$

where *A* is the volume segmented by an expert in obstetrics and *B* represents the automatically segmented volume. Additionally, the Hausdorff Surface Distance (HSD) [39] and the area under the receiver operating characteristic (ROC) curve (AUC) were obtained. After segmentation validation, the RF was applied to the remaining studies.

Subsequently, to validate the proposed PL-CPD method, the Target Registration Error (TRE) (equation (16)) between the reference volume *V*_*R*_, and the study to be aligned *V*_*M*_ were measured considering the eighteen pairs of registered volumes. A total of *n* = 1000 uniformly distributed target points were defined as *a*_*i*_ and *b*_*i*_, corresponding to the registered and reference volumes, respectively:

(16) $$\begin{array}{c} \text{TRE}\left(V_{R},V_{M}\right)=\sqrt{\frac{1}{n}\overset{n}{\underset{i=1}{\sum }}{\left(b_{i}-a_{i}\right)}^{2}}. \end{array}$$

According to Fitzpatrick [40], TRE provides a better estimation of registration errors than a landmark-based metric. Additionally, to evaluate in detail translation and rotation errors, the Root Mean Square (RMS) difference was used. It can be denoted as follows:

(17) $$\begin{array}{c} \text{RMS}\left(V_{R},V_{M}\right)=\sqrt{\frac{1}{m}\overset{m}{\underset{k=1}{\sum }}{\left(b_{k}-a_{k}\right)}^{2}}. \end{array}$$

To measure displacement errors, the values in the three axes (Δ*X*, Δ*Y*, and Δ*Z*) are considered as *b*_*k*_ and *a*_*k*_ with *m*=3; on the other hand, for the rotation errors the values of the angles (*α*, *δ*, and *λ*) are taken as *b*_*k*_ and *a*_*k*_.

To evaluate its performance, the proposed PL-CPD algorithm was compared with other previously reported intensity-based registration methods: Mutual Information (MI), Cross Correlation (CC), and Mean Squared Error (MSE) [6–8]. The method was also compared with some geometric registration methods based on correspondence, such as Iterative Closest Point (ICP) [12, 13], Random Sample Consensus (RANSAC) [14], Scale-Invariant Feature Transform (SIFT) [15], and Coherent Point Drift (CPD) [16]. For all comparisons, TRE's mean and standard deviation were obtained from the accumulated errors measured for each registration case. Statistical differences for these tests were determined by an paired Student *t*-test (*p* < 0.05), comparing the PL-CPD method with each other. In addition, the mean RMS errors for translations and rotations were obtained for each carried out registration.

## 3. Results and Discussion

**Figure 3:**
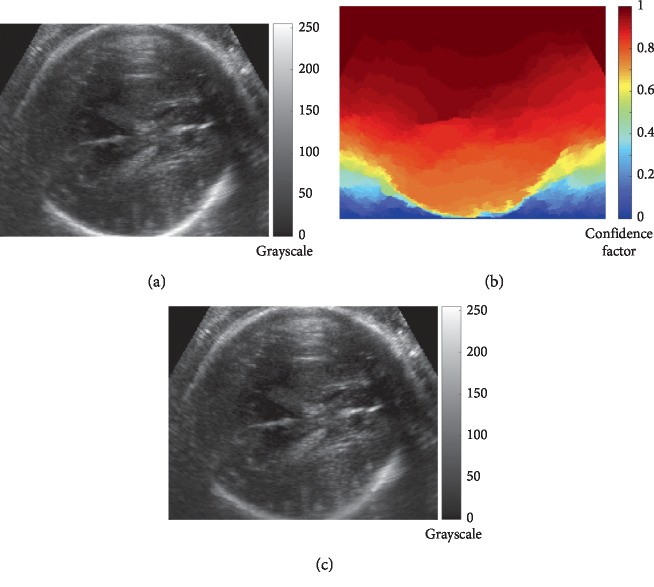
Example of the US images' weighting using confidence maps: (a) representative fetal head US image in axial view, (b) corresponding confidence map, and (c) weighted image.

In this section, results of the proposed registration method (PL-CPD), as well as the corresponding discussion are presented. As mentioned in Section 2.2, the procedure begins with a preprocessing step of background normalization with confidence maps. An example of the results obtained is shown in Figure 3: fetal head image in axial view (Figure 3(a)), its corresponding confidence map (Figure 3(b)), and the image obtained after normalization (Figure 3(c)). It can be noted that the weighted image shows less intensity variation, specifically in those regions with the lowest contribution of the confidence map (yellow and orange areas). As a result, intensity variations around the fetal head are attenuated as can be seen by comparing the images before and after confidence map weighting.

To evaluate the performance of RF classification, five labeled fetal head volumes were used, where the task was to discriminate between voxels corresponding to the fetal cranium (class *c*=+1) and the rest of structures (class *c*=−1). For this, 5-fold crossvalidation was used. Segmentation results for the five evaluated volumes are presented in Table 1. It can be seen that all metrics show consistent results with standard deviations of 3.5, 1, and 3.4% for the Dice, HSD, and AUC indices, respectively. In addition, the average performances obtained by Dice and AUC metrics are higher than 80%, which is considered acceptable to generate the point clouds and the probabilistic maps needed in the registration process.

**Figure 4:**
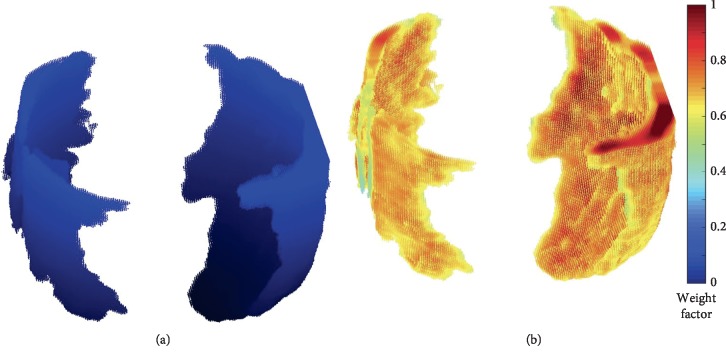
Examples of fetal head point clouds models: (a) set of points without weighting and (b) the same point cloud with probabilistic weights denoted by colors.

In Figure 4(a), an example of fetal cranium segmentation is presented, where it can be seen that it contains several missing regions due to US acoustic shadows. As already mentioned, these missing data hinder the alignment process. In addition, Figure 4(b) shows an example of the same point cloud with the weighting factors obtained using RF. It can be noticed that the distribution of weights is not uniform, due to the fact that not all the points have the same probability of belonging to the fetal cranium. As detailed in the methodology section, these weights are used as membership probabilities of the GMM used in the CPD method.

**Figure 5:**
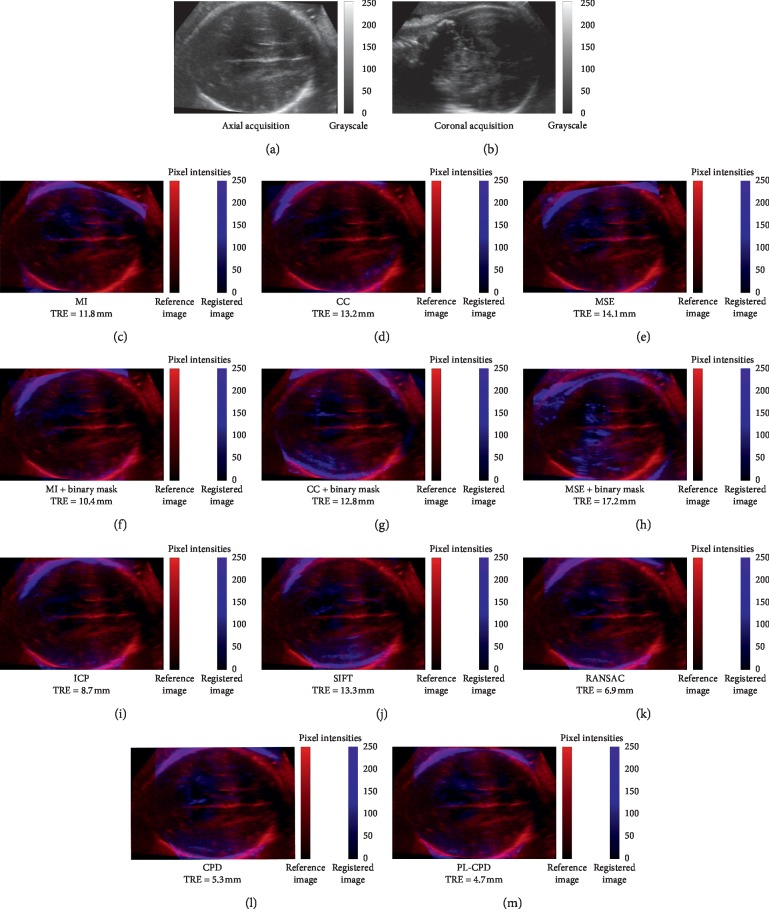
Representative example of each method's registration results using two volumes in axial (a) and coronal (b) view. The reference volume (axial acquisition) is shown in red and the aligned volume (coronal acquisition) in blue.

The visual outcome of each method's registration is shown in Figure 5. Volumes to be aligned were acquired in an axial and coronal view (Figures 5(a) and 5(b), respectively). To better appreciate registration results, the reference volume is shown in red (axial view) and the aligned volume is presented in blue (coronal view). In all cases, because both registered volumes contain different and complementary information, differences in the visual results of the registration are shown. However, a good alignment result can be seen in structures such as cranial circumference and ventricles. In the second row, results obtained with intensity-based methods are shown, where the best performance corresponded to the MI algorithm. These same methods combined with a binary mask provided the registrations presented in the third row (Figures 5(f) and 5(h)). It can be noticed that MI and CC improved when using the binary mask, contrary to MSE. The fourth row of Figure 5 shows the results of geometric-based methods, where RANSAC presented the best performance, observed with a better adjustment of the fetal cranial circumference. Finally, in the last row, a comparison of the CPD method (Figure 5(l)) with the proposed method (PL-CPD) (Figure 5(m)) can be appreciated. It can be noted that performance measured by TRE of PL-CPD is better compared to CPD. The corresponding registered images show a good alignment of fetal heads and of the cerebral ventricles' central region.

**Figure 6:**
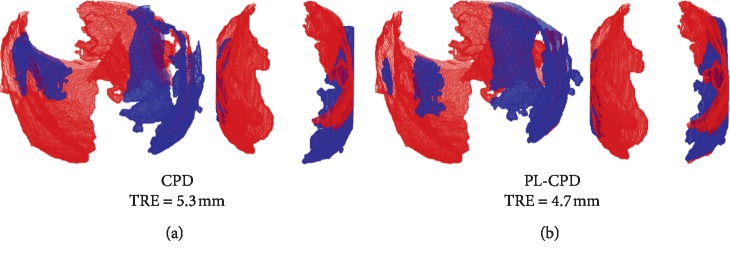
Representative example of the correspondence between a pair of aligned fetal head points cloud. (a) Using the CPD method and (b) with the proposed method (PL-CPD).

Visual results of the registered point clouds using CPD and PL-CPD methods can be seen in Figure 6: in red the fixed set of points and in blue the aligned points cloud. It can be observed that the PL-CPD method shows a slight improvement in aligning both studies (Figure 6(b)) compared to the CPD method (Figure 6(a)). This can be due to the contribution of the nonuniform assignment membership probabilities of the GMM.

To perform a quantitative evaluation of the obtained results, as well as a comparison with other registration methods commonly used in the literature, the TRE was calculated between the reference volume and the study to be registered. The results are shown in Table 2. To determine the statistically significant differences between the proposed and the other methods a paired Student *t*-test (*p* < 0.05) was carried out for each comparison. The first section of Table 2 shows the results in millimeters of several intensity-based algorithms, where MI presented the smallest error followed by MSE and CC; all of them showed statistical differences compared with the proposed method. As expected, these methods, based entirely on intensity information present considerable errors when aligning US fetal brain studies with acoustic shadows and artefacts that alter the registration result.

As described in the methodology section, the intensity-based methods were combined with a binary mask product of fetal head segmentation. In this way, registration was carried out only with visible information, without considering acoustic shadows. The results are shown in the second section of Table 2. A general improvement can be observed in each method obtaining a 78% TRE reduction for MI-BM, 81% for CC-BM, and 92% for MSE-BM, compared with their corresponding versions without using a binary mask. Despite these improvements, these three methods present statistical differences with respect to the proposed method. Binary masks helped to attenuate artefacts introduced by acoustic shadows in the registration process, but because both registered studies do not have the same information, the alignment result is affected.

In the last section of Table 2, results obtained with geometric-based methods are presented, of which ICP (9.78 ± 4.65 mm), SIFT (10.28 ± 4.83 mm), and RANSAC (7.73 ± 4.57 mm) show statistical differences compared to PL-CPD. In contrast, CPD presents a small error with 6.89 ± 4.08 mm. Finally, the result obtained with the proposed method shows the best performance of all with 6.38 ± 3.24 mm, which outperforms the CPD algorithm. This indicates that the incorporation of probabilistic weights as probability of belonging contributes to the alignment process.

Additionally, computing times for each of the compared algorithms were measured and are shown in the last column of Table 2. The first section shows that intensity-based methods present the highest times, with 37.5 ± 9.3 min for MI, 49.9 ± 13.2 min for CC and 39.6 ± 16.1 min for MSE computations. When binary masks are incorporated to these algorithms, times are considerably reduced down to 56.7%, 51.9%, and 56.6% for MI-BM, CC-BM, and MSE-BM, respectively. In contrast, computation times for geometry methods are inferior to those obtained by intensity-based strategies, being 16.4 ± 8.8 min for RANSAC, followed by SIFT with 14.3 ± 6.2 min. The PL-CPD algorithm shows an average computational time of 12.7 ± 4.8 min, which represents an increase of 4.7% with respect to CPD. Finally, ICP took the best computation time, with 9.5 ± 2.7 min, but it also presents considerable registration errors. These results concur with a similar evaluation reported by Savva et al. [11], where it is concluded that intensity methods are heavier in computational cost because of a wider search space for optimal registration. Another aspect to be considered is that several of the compared correspondence-based and intensity-based methods combined with binary mask (marked with + in Table 2) require a previous fetal cranium segmentation with RF, which adds 7.4 ± 2.6 min to overall computation times. Furthermore, given that RF is a supervised classifier, it requires a previous training step (described in Section 2.2) that represents an average time of 12.8 ± 3.4 min. This step is carried out only one and therefore it is not included in computational times reported in Table 2.

In a preliminary study, an SVM classifier to segment fetal cranium was proposed. The TRE obtained by SVM was 6.21 ± 3.78 mm, which is similar to the TRE shown by RF; however, the average processing time by SVM is 20.9 ± 4.8 min, which is 60.8% greater than RF.

All evaluations were carried out in MATLAB2019a with a 6 Core™ Intel® i5-8500 processor at 4.1 GHz and a DDR4 memory at 2.6 GHz of 4 Gb.

**Figure 7:**
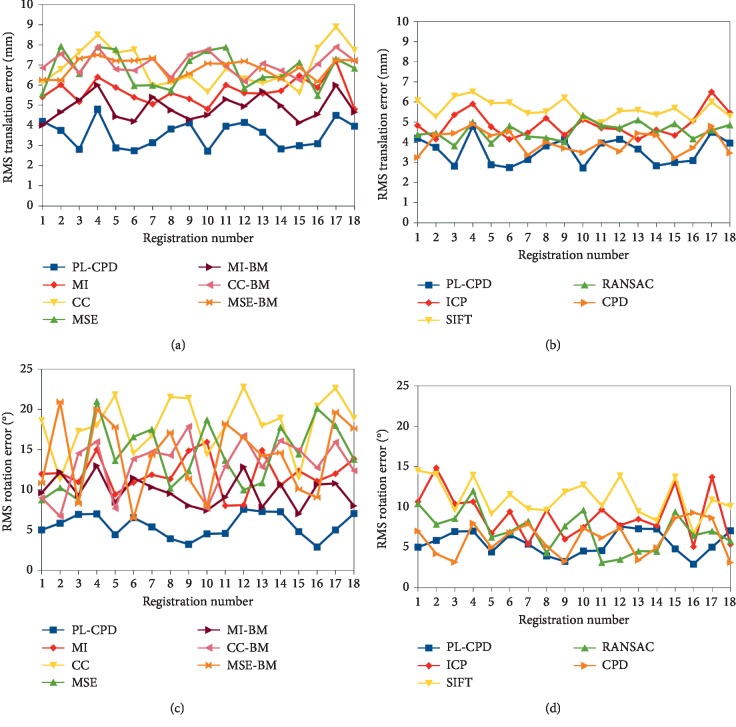
RMS registration errors of each method. In the first row, translation errors of intensity and geometry-based methods are presented (a and b, respectively). The corresponding rotation errors are shown in the second row (c and d).

The detailed translation and rotation errors for each pair of registered studies can be seen in Figure 7. Comparison of translation errors obtained with the proposed PL-CPD method and intensity-based methods with and without binary masks is presented in Figure 7(a). It can be noticed that in all cases our method provides lower errors, except in the first case registration where MI-BM shows a slightly better result. In contrast, translations errors obtained with geometric methods are shown in Figure 7(b). It can be observed that the PL-CPD method presents better results in most cases. Only RANSAC algorithm exceeds PL-CPD in one of the alignments (case 9) and the CPD method exceeds PL-CPD in four cases, regarding translation.

On the other hand, rotation errors measured in degrees are presented in Figures 7(c) and 7(d). In the former, performances of intensity-based methods show that there is a high variability. This phenomenon may be due to acoustic shadows and missing information on the studies. It can be noted that in all cases the PL-CPD method is more stable and provides lower errors, except for subject number six where it is comparable to the MSE-BM approach. When contrasting our method with other geometry-based methods (Figure 7(d)), it can be seen that rotation errors are competitive, only outperformed in all cases by ICP and SIFT algorithms, which is consistent with what is reported in Table 2. Comparing RANSAC vs PL-CPD it can be seen that the proposed method is exceeded in five pairs of volumes. Finally, when contrasting CPD vs PL-CPD methods it can be noticed that the latter is surpassed in only six pairs of alignments. This reflects that the proposed method incorporating results of RF voting process as the GMM membership probability helps to attain a finer adjustment in the alignment process.

Additionally, it can be observed that the incorporation of binary masks boosted intensity-based methods' performance, by only considering information of the segmented head during registration. Also, it must be noted that in case number four and seventeen the volumes presented several occlusions which are reflected in higher rotation and translation errors for most of the tested methods. In general, the proposed method presents better results and less variation compared to other intensity and geometry based methods.

It should be considered that US volumes registered in this research have a high percentage of occlusions, noise, and missing data, unlike applications previously reported by other authors, where their data do not contain noise or occlusions. On the other hand, until now all reported variants of CPD use point clouds previously constructed. In contrast, we propose an approach that builds the cloud of points from the fetal cranium segmentation and at the same time estimates the values of posterior probability incorporated as probability weights of nonuniform membership of the GMM.

In the context of aligning US data, the errors obtained from the proposed method are comparable with those reported by Fathima et al. [29], who obtained a TRE of 3 ± 1 mm in US studies. However, these authors did the registration using 2D studies and without any artefacts. They mention that a gestational second-trimester fetus has a cranial circumference going between 15–30 cm and a biparietal diameter of 4.5 to 6 cm; therefore, a TRE in the range of millimeters may be acceptable in clinical practice. Other works have carried out the registration of US fetal brain studies in 2D and 3D. However, they have limitations such as registering in a single US study with itself and without considering occlusion artifacts [27, 28] or registering phantoms where conditions are controlled and there are no occluded areas [30].

As mentioned before, unlike the previously reported studies, the proposed method deals with the problem of US brain volumes registration with a considerable degree of occluded areas and noise, phenomenon that occurs in real cases mainly in second and third pregnancy trimesters. It considers that the proposed PL-CPD method may be useful in the assessment of fetal growth, to segment several internal brain structures or to combine different US fetal head acquisitions [4].

## 4. Conclusions

We presented a novel scheme for the automatic registration of real 3D US fetal brain volumes, volumes that contain a considerable degree of occlusion artefacts, noise, and missing data. To the best of our knowledge of the literature, there are not any reported 3D alignment techniques using real fetal images with these characteristics.

To register, a new algorithm named PL-CPD is herein proposed, which incorporates two new features worth highlighting: automatic segmentation and conformation of the point cloud, qualities that have not been reported by any previously developed CPD variant, together with an appropriate proposed weighting of membership probabilities of the GMM. The segmentation and the weighting factors were obtained from a supervised learning algorithm based on a RF classifier and fed by a set of features composed of intensity, texture, and edge patterns.

This weighting helps to attain a finer adjustment during the aligning of US data because not all points have the same membership probabilities. The PL-CPD method can work with point clouds contaminated with outliers, missing data, or multiplicative noise; these characteristics mean that PL-CPD can adequately register fetal brain US volumes, even in the presence of acoustic occlusions and speckle noise.

When comparing the PL-CPD algorithm with other intensity-based methods we obtained a better performance; this may be due to the fact that intensity-based methods are affected by acoustic occlusions, and therefore the studies to be aligned do not share the same information, which complicated an adequate correspondence. In comparison with other geometry-based methods, PL-CPD preserves a lower global error (TRE) and less deviation in translation and rotation. This may be due to the incorporation of nonuniform membership probabilities to the GMM.

The developed PL-CPD algorithm can be useful in clinical practice in one or all of the following cases: to quantify fetal brain growth and development, to monitor the evolution of indicators such as biparietal diameter or cranial circumference, to segment different structures using a fetal brain template, and to align and combine multiple 3D US fetal brain volume acquisitions. The method can also be applied to other medical image registration problems, provided that the Random Forest classifier can be adequately trained.

## Figures and Tables

**Table 1:** Quantitative evaluation of fetal head segmentation using RF classifier.

No.	Dice (%)	HSD (mm)	AUC (%)
1	91.3	4.8	88.2
2	86.7	5.7	82.4
3	88.6	5.1	85.4
4	85.4	6.6	83.5
5	82	7.3	79.2
Global (*μ* ± *σ*)	86.8 ± 3.5	5.9 ± 1	83.7 ± 3.4

**Table 2:** TRE and computational times of different registration methods (*μ* ± *σ*).

Registration method	TRE (mm)	Computational time (min)
*Intensity-based*		
Mutual information (MI)	12.47 ± 4.1^*∗*^	37.5 ± 9.3
Cross correlation (CC)	16.23 ± 6.77^*∗*^	43.9 ± 13.2
Mean square error (MSE)	15.20 ± 7.53^*∗*^	39.6 ± 16.1

*Intensity-based* *+* *binary mask*		
Mutual information (MI-BM)	9.74 ± 4.03^*∗*^	21.9 ± 6.2^+^
Cross correlation (CC-BM)	13.10 ± 7.25^*∗*^	22.8 ± 4.1^+^
Mean square error (MSE-BM)	13.98 ± 7.31^*∗*^	22.4 ± 5.3^+^

*Geometric-based*		
Iterative closest point (ICP)	9.78 ± 4.65^*∗*^	9.5 ± 2.7^+^
SIFT	10.28 ± 4.83^*∗*^	14.3 ± 6.2
RANSAC	7.73 ± 4.57^*∗*^	16.4 ± 8.8^+^
Coherent point drift (CPD)	6.89 ± 4.08	12.1 ± 5.1^+^
Probabilistic learning-CPD (PL-CPD)	**6.38** ± **3.24**	12.7 ± 4.8^+^

^*∗*^Statistically significant differences with respect to PL-CPD method (*p* < 0.05). ^+^These values include prior RF fetal cranium segmentation necessary to generate the binary mask and the point cloud.

## Data Availability

The data used to support the findings of this study are available from the corresponding author upon request.
